# Prof. Charcot and His Experiments at La Salpétrière

**Published:** 1879-02

**Authors:** W. S. Caldwell

**Affiliations:** Berlin


### Foreign Correspondence.

### ARTICLE XII.

### PROF. CHARCOT AND HIS EXPERIMENTS AT LA SALPÉTRIÈRE.

I brought with me from America a letter of introduction to Prof. Charcot, and visiting the Salpétrière soon after my arrival in Paris, in August, 1877, was fortunate in presenting it. At the same time an English physician of considerable eminence had called on the renowned neurologist, and we thus had the opportunity of seeing several of his demonstrations in private.

Although his experiments and their apparent results seemed to me something remarkable and inexplicable, I did not at the time, attribute much importance to them from a practical point of view, especially as Prof. Charcot was himself, *then*, undecided as to whether or not his new theories would develop to the point of utility in the treatment of these cases.

When I returned to Paris in the Autumn of 1878, and began attending Prof. Charcot's regular lectures at La Salpétrière, I found him more enthusiastic than ever regarding his new facts, which he went on to demonstrate in several discourses.

Such interest has the subject of his lectures elicited, that I saw among his attentive listeners, such men as Virchow and Martin, of Berlin, and many other renowned men of our profession. Not only on the Continent, but in Great Britain, his teachings are attracting so much interest, that I am induced to give the following summary of them, to the exclusion of matters which, I, myself, consider of more practical interest. Though it is Prof. Charcot upon whom, as a public teacher, the interest in this subject is at present directed, it was a Dr. Burge who claims to have made the first discoveries in this direction. The latter first published his views, I believe, in 1876.

On my first visit to La Salpêtrière I was shown two female patients, one about eighteen and the other twenty-three years of age, who were both the victims of frequent attacks of what Prof. Charcot denominates "Hystero-Epilepsy." One of these patients, the younger, had had these convulsions for the last two years very frequently, sometimes even to the number of fifty and one hundred in the twenty four hours. In spite of all this her intellect was not in the least affected; and that fact, in connection with this, that no period of cerebral congestion followed the fits, as in true epilepsy, are valuable aids in making a differential diagnosis between the two diseases.

These girls were both completely hemi-anæsthetic. On the side that was insensible, a large darning-needle could be passed through the skin without the least sensation being perceived by the patient.

### METALLO-THERAPEUTICS.

But the most wonderful of the phenomena in these cases, was that by the application of certain metals or a large horse-shoe magnet to the side that was deprived of sensation, this abnormal condition would change from the side to which the metal was applied, to the one that had been before entirely normal. In one of these patients, who was anæsthetic on her *left* side, Prof. Charcot applied a twenty-franc gold piece to the outer aspect of the left arm, and after the expiration of three minutes it was found that a small circle around the gold coin had regained its sensibility, while at the same time a corresponding surface on the *right* arm had lost all sensation.

At the end of ten minutes the patient was completely anæsthetic on her *right* side, and had regained sensibility on her *left*.

Prof. Charcot said that some persons were susceptible to one metal and some to another, and brought forward the second patient in whom he produced exactly the same phenomena as in the first, by using a copper plate instead of a gold coin. He also stated that in all these cases there existed an ovarian tenderness on the side that was anæsthetic.

But the practical part of this matter as claimed by both Dr. Burge and Prof. Charcot is, that the metal to which the patient is susceptible, or in other words, that which will change the anæsthetic condition from one side to the other, is the remedy that is indicated in the treatment of each particular case.

To the patient who was sensible to gold, he was giving daily .02 gramms of the chloride of gold and sodium.

To the patient who was sensible to copper, he was giving .3 grams of the hydrated binoxide of copper three times in a day. Through last year Prof. Charcot said he had not used these remedies sufficiently long to test their efficacy ; this year in his public lectures he declares that the medicines have been in each case highly beneficial in lessening the frequency of the attacks, and improving the general condition of the patients. Still, he did not admit that in any instance he had been able to effect a complete cure.

Although the principles involved were the same as in the experiments I had seen Prof. Charcot perform in private, in his public lectures he greatly extended them, and brought forward a number of new cases to illustrate his subject.

In one case the entire throat was so entirely anæsthetic that the Doctor could irritate the epiglottis with his finger without producing the slighest reflex phenomenon.

A copper plate, 3 by 5 centimeters, was tried over the front of this patient's throat for seven minutes, at the end of which time she was so sensitive that any attempt at passing the finger into the pharynx brought on violent attempts at vomiting. The hystero-epileptic patients who were hemi-anæsthetic had many of them another remarkable symptom, and that was complete color-blindness on the side that was insensible.

In the case of a girl eighteen years of age who was anæsthetic on her right side, the left eye was closed, and red, blue, green and orange-colored cards that were passed before the right eye, were all pronounced by the patient as *white*.

The copper plate that had been bound on to the throat of the previous patient, was bound on to the right temple of this one, and in five minutes she could accurately discern colors with her right eye, but was as completely color-blind in her left, as she had previously been in her right. The migration of sensibility and sense that these persons experienced upon the application of the metals was, in every instance, only temporary in character, as, after the expiration of a variable period of time, say from ten to thirty minutes, the old condition of hemi-anæsthesia was found to exist on the side at first affected.

A woman, 47 years of age, who had passed the menopause was presented, who had been the victim of hystero-epileptic attacks from her early womanhood, a case demonstrating the fact that this disease will not always cease with the cessation of the menses.

This patient had had for years a permanent contraction of the flexors of the fingers and hand of the right side. A large horse-shoe magnet was placed near the outer aspect of the right arm, and in about five minutes the rigid contractions in the muscles of this side began to give way, and in ten minutes she was able to use freely and without restraint the right hand, but the condition of contraction had been transferred to the flexors of the left hand.

The transposal of this abnormal condition only lasted for a few moments after the magnet was removed, for in fifteen minutes her right hand presented the same appearance as when she entered the room.

During his second lecture Prof. Charcot brought on to the platform two patients whom I recognized at once as those upon whom I had seen him experiment in 1877. They were seated in chairs near each other; and an immense tuning fork placed between them. This instrument was then struck, and when it began to sound, both girls with perfect accord fell into a cataleptic condition. Their eyes widely opened seemed to gaze with a vacant stare at some far-off object. Their hands, arms, and other parts of their bodies could be placed in any given attitude and would thus remain for an indefinite period of time. They could be aroused from this condition by suddenly blowing into their faces, or pressing upon the ovary that was sensitive.

The sudden flame of a light from an electric lamp thrown into the eyes of these patients, or even the fixed gaze of Prof. Charcot's eye, would produce the same phenomena. From this cataleptic condition, if not disturbed, the patient would pass into that of a somnambulic state. Prof. Charcot said: "This is the condition into which animal magnetizers plunge hysterical persons, and, during its persistence, they cause them to perform mystical acts such as walking in the dark, describing absent persons, distant places, and the like. From this condition I arouse this patient by pressing suddenly upon one of her ovaries. She may not be entirely herself for a quarter of an hour, but will soon regain her normal condition."

In his third lecture Prof. Charcot brought into the lecture-room a girl about 20 years of age lying upon a cot, dressed in a long garment, the sleeves and legs of which extended far beyond the hands and feet, and to which were attached long pieces of cord to control her during her convulsive paroxysms. The patient had an aparatus around her body (a new invention of Dr. Poirier), constructed something like a tourniquet for compressing the abdominal aorta, but in which the pad was made to press upon the right ovary. This truss was removed from this patient, and she at once went into a series of violent convulsions. During the time of these attacks Prof. Charcot gave a minute description of each phase of her symptoms.

As to the diagnosis he said: "Of the affection, as distinguished from true epilepsy, we have first to observe that the fits always occur during the waking hours of the patient.

"Again, they never fall down in the street in convulsions, for they have always premonitory symptoms that last a sufficient length of time to allow them to seek a place of safety before the attacks occur.

"Each paroxysm consists of a certain regular and definite series of evolutions.

"First, you see this patient extends her arms over her head, then gradually draws them down over her abdomen, and, after keeping them in a tonic spastic condition of contraction for a few seconds, she begins a series of active clonic spasms.

"This stage in which the patient is at present, is the epileptiform period of the attack.

"There is no physician of experience who, stepping into this room at this moment and viewing this patient for the first time, but would diagnosticate her case as one of *true* epilepsy.

"Our patient, after these clonic spasms are over, will remain quiet for a few seconds, and then the attack will resume its hysterical type again, *phase des grands mouvements*, in which you see she executes a series of rapid to and fro movements with her entire body. When these *grands mouvements* are over, the next stage of the attack begins, which is *la phase des attitudes passionelles*, in which, raising herself to a sitting posture, she alternately strikes an attitude and wears an expression of fear and hatred, and then of joy and extreme bliss.

"At one time she will see horrid objects that approach her, and in the next moment her visions are of the most fascinating kind, and she hears the strains of sweetest music.

"After these last phenomena have occurred, our patient will remain quiet for an indefinite period of time, and will then execute again the same series of movements and attitudes as before."

After the doctor had allowed her to undergo these convulsions sufficiently long to illustrate to his hearers all their different stages and symptoms, he pressed firmly with his closed fist on her right ovary, and the patient, heaving a long sigh of relief, the convulsions ceased at once.

The instrument for the production of continuous pressure was replaced, and in five minutes she was again in a normal condition.

The hystero-epileptic attacks with these patients usually occur without immediate exciting cause, although fear and anger have a tendency to develop them.

There is, in most of them, an *epileptogenic zone*, which may be situated in any part of the body, and which, being pressed upon, or in any way irritated, will throw the patient at once into a violent series of convulsions.

Prof. Charcot, just before the close of one of his lectures, showed a person in whom he said this sensitive zone was at the last cervical vertebra.

As the girl stood in the hall through which the students passed as they went from the lecture-room, one of them took occasion to verify Prof. Charcot's assertion by suddenly pressing his finger on this point of her spine. The poor creature fell down as suddenly as if shot, on the stone floor, and went into convulsions of as violent a character as I ever saw.

Dr. Oulmont, Prof. Charcot's interne, soon made his appearance, and coming down with the whole weight of his body upon the right ovary of the patient with his closed hand, he was soon able to arrest the paroxyms.

Prof. Charcot closed this lecture by showing us, by means of a magic lantern, copies of paintings of the old masters, representing persons as being possessed of the devil, and priests casting them out, in whom the attitudes and expressions of the persons represented were an exact counterpart of some phase or stage in the hysterical attacks of the patients he had shown us.

WHAT IS THOUGHT OF PROF. CHARCOT AND HIS TEACHINGS.

A vast majority of those who listen to Prof. Charcot at La Salpétrière have such an implicit faith in the integrity and wisdom of the man, that they believe that he could not be deceived by the patients upon whom he operates, and would present nothing to his hearers in which he *himself* had not the most unbounded faith.

The great Claude Bernard evidently believed these phenomena to be genuine, for, after witnessing them just before his death, he was perfectly astounded at their manifestations, and exclaimed, "*these facts show us how little we know.*"

Although this represents the sentiments of that portion of the profession who are thrown in immediate contact with Prof. Charcot, it is far from being generally adopted outside of Paris and France.

Here, in Berlin, the first evening that I went to hear Prof. Westphal, he brought out for the subject of his lecture an hystero-epileptic patient.

This patient was fifty-three years of age, and was what Prof. Westphal designated as a true type of hystero-epilepsy which has been of twenty-five years duration. She was nearly idiotic, thus showing that the disease *does* effect the intellect.

He claimed that the nice points in the diagnosis of this disease as given by Prof. Charcot, could not always be recognized in practice.

A case like this might be one of *genuine* epilepsy affecting a person of hysterical temperament, in which one could not always decide, as to where, in any given attack, the hysterical element ends, and the epileptic begins.

He went on to refute the generalized statement that one could arrest the paroxysims by pressure upon the ovaries, so that ovarian tenderness was a prominent symptom attending them.

He said that he and Prof. Schröder had experimented upon a number of cases by making *direct* pressure upon the ovaries by combined manipulation through the vaginal and abdominal walls and had found that the ovaries were not generally in the least sensitive. Besides he was of the opinion that Prof. Charcot, with his mode of manipulating, *did not in one case out of five touch the ovary.*

Furthermore in the single case of a male patient, whom he had had under observation and who had all the symptoms of genuine hystero-epilepsy, he was able to arrest the paroxysms as effectually by firm pressure into one of the iliac fossæ as Dr. Charcot had been able to do in his cases, where he had *supposed* he was pressing upon a sensitive ovary.

He believes that the arrest of the convulsive attacks in these cases is due to a general reflex action produced by pressure upon the pelvic nerves.

After this lecture, I had a private interview with Prof. Westphal, and he, knowing that I had just came from Paris, began at once to question me as to what I had seen of Prof. Charcot's doings at La Salpêtrière. Finding him skeptical as to the genuinenesss of the entire phenomena of metallo-therapeutics I asked him his explanation of Prof. Charcot's experiments.

He told me then promptly that, much as he had formerly admired the great French physiologist, in this matter, Prof. Charcot was playing the part of a mountebank and juggler. I then asked him what object a man of his standing could have for taking such a course. "Money! money!" was his reply. At this juncture we were interrupted or I should have interrogated Prof. Westphal farther; for, to me, it is inconceivable that a man of Prof. Charcot's standing should take the course in this matter that he does, unless he firmly believes in what he teaches; besides, I cannot see how he can make money from his demonstrations and lectures, as all of them are free.

As bearing upon this subject and giving, in my view, the best explanation I will close the article by giving the following history of a case as published by Dr. Hughes Bennett, in a late number of the *British Medical Journal:*

In August, 1877, a woman 44 years of age, was admitted to Dr. Bennett's wards, having for four years been the subject of a variety of nervous attacks, without real convulsions. She was in tolerable health but was entirely anæsthetic on her right side. Needles passed through the skin of the side, and the most powerful Faradic currents produced no sensation. The left side was nearly natural.

From Aug., 1877, to April, 1878, the patient was treated by tonics and electricity, but without any effect; in fact the paresis and anæsthesia had become more marked. The attention of Dr. Bennett having been called to the subject of metallo-therapy by some published accounts of Prof. Charcot's demonstrations, he (Dr. B.,) resolved to put his patient upon this treatment.

Accordingly the patient was blindfolded, so that she could not see what was doing, and discs composed of various metals as gold, silver, copper, lead, zinc, and tin were applied to the anæsthetic side without producing the least effect.

After Dr. Bennett had tried these experiments repeatedly, and was convinced that no result would follow, he invited several medical friends to witness them. At this time he applied a zinc plate to the arm, and after it had been on for twenty minutes the patient said she felt a tickling sensation in the part, and, to the Doctor's surprise, he found that slight pricks of a pin could be felt. A few moments later, sensibility had returned to the side so long insensible. Twenty-four hours after this experiment, the right side was found in its original anæsthetic condition.

It was now found that *all* the metals that had before failed to produce an effect upon this patient, would each, when applied, produce a temporary return of sensibility to the parts. At this juncture Dr. Bennett applied two discs of wood upon the arm, and found that after they had been on for a few moments, sensibility had returned to the side exactly as when *metals* had been used.

The result of all this was, no marked benefit to the patient, and some months later she was sent to the seaside. Dr. Bennett deduces from this, that the reason why he produced no effect upon his patient in his first experiments was that she was entirely ignorant of what results were attempted to be attained.

When he had summoned his friends in order to demonstrate to them that Prof. Charcot's results were not obtainable, lo, the very phenomenon that he was positive would *not* occur, did actually happen; and why? Simply because it was necessary for him to make some explanation of the subject in the presence of his patient. As soon as she knew what *effect* was intended to be produced, that effect followed.

Dr. Bennett closes his review of this subject in these words: "There are always those who, either from an involuntary bias of mind, or from a natural credulity in what appears marvelous, seem to think that what is difficult of explanation must of necessity be true. Thus does the spiritualist and the electro-biologist pledge his faith to what his reason cannot comprehend, and what he perhaps honestly believes to be genuine.

"As well might one credit the reality of the tricks of a conjuror, because it baffles all our efforts to appreciate the steps by which he deceives us.

"In all these hypotheses, the more we eliminate sources of error, the more that unprejudiced observers investigate the subject, and the more that stringent tests are applied, the more easy and simple do we find these 'inexplicable phenomena' become.

"It may be that metallo-therapy, the most recent of the doctrines which have flattered the psychological world, is doomed to an early grave, to be resurrected in the future in some other novel and more attractive form." W. S. Caldwell.

Berlin, Jan. 1, 1879.

---

Diphtheria was named from the skin of the goat Amalthea, on which Jove wrote the destiny of man.

